# Overexpression of SHMT2 Predicts a Poor Prognosis and Promotes Tumor Cell Growth in Bladder Cancer

**DOI:** 10.3389/fgene.2021.682856

**Published:** 2021-06-04

**Authors:** Peng Zhang, Qian Yang

**Affiliations:** ^1^Department of Urology, Guizhou Provincial People’s Hospital, Guiyang, China; ^2^Department of Gastroenterology, Guizhou Provincial People’s Hospital, Guiyang, China

**Keywords:** SHMT2, bladder cancer, prognosis, cell cycle, apoptosis

## Abstract

SHMT2 was overexpressed in many tumors, however, the role of SHMT2 in bladder cancer (BLCA) remains unclear. We first analyzed the expression pattern of SHMT2 in BLCA using the TNMplot, Oncomine, the Cancer Genome Atlas (TCGA), and the Gene Expression Omnibus (GEO) databases. Next, the association between SHMT2 expression and overall survival (OS)/disease-free survival (DFS) in BLCA patients were analyzed using TCGA and PrognoScan database. The correlation between SHMT2 expression and clinicopathology was determined using TCGA database. Furthermore, the genes co-expressed with SHMT2 and their underlying molecular function in BLCA were explored based on the Oncomine database, Metascape and gene set enrichment analysis (GSEA). Finally, the effects of SHMT2 on cell proliferation, cell cycle, and apoptosis were assessed using *in vitro* experiments. As a results, SHMT2 was significantly overexpressed in BLCA tissues and cells compared to normal bladder tissues and cells. A high SHMT2 expression predicts a poor OS of BLCA patients. In addition, SHMT2 expression was higher in patients with a high tumor grade and in those who were older than 60 years. However, the expression of SHMT2 was not correlated with gender, tumor stage, lymph node stage, and distant metastasis stage. Finally, overexpression of SHMT2 promoted BLCA cell proliferation and suppressed apoptosis, the silencing of SHMT2 significantly inhibited BLCA cell proliferation by impairing the cell cycle, and promoting apoptosis. SHMT2 mediates BLCA cells growth by regulating STAT3 signaling. In summary, SHMT2 regulates the proliferation, cell cycle and apoptosis of BLCA cells, and may act as a candidate therapeutic target for BLCA.

## Introduction

Bladder cancer (BLCA) ranks nine as a cause of cancer-related mortality worldwide and is the second most common malignancy of the human urinary tract. In 2020, there will be approximately 81,400 new cases of urinary bladder carcinoma diagnosed and 17,980 cancer-related deaths (Siegel et al., 2020). Approximately 75% of BLCA cases are non-muscle-invasive, while approximately 25% are muscle-invasive (Kamat et al., 2016). Because of the completely different biological characteristics, there are large differences in disease progression, overall survival, and treatment (Berdik, 2017). The main treatment for the latter is radical cystectomy, which has high postoperative recurrence, distant metastasis rates, and a low postoperative 5-year survival rate (Ploussard et al., 2014). Hence, identifying biomarkers that are useful in the early diagnosis, treatment, and prognostic assessment is necessary to improve BLCA outcomes.

Tumor growth is a complex process, cell proliferation is tightly modulated by the cell cycle, and an impaired cell cycle may suppress cell proliferation and tumor growth. Mitochondrial function alteration is an important step that is involved in the physiopathology of cancer (Minton et al., 2018). Furthermore, mitochondria are the regulatory centers of cellular energy metabolism.

The serine hydroxymethyltransferase enzyme (SHMT) contains two members, SHMT1 and SHMT2. SHMT2 is the main enzyme that converts the serine to glycine in mitochondrion and acts as a key source of glycine in proliferating cells (Ahn and Metallo, 2015; He et al., 2019). Previous studies showed that SHMT2 encodes the mitochondrial form of a pyridoxal phosphate-dependent enzyme, which is required for energy metabolism (Minton et al., 2018). In this study, the molecular functional analysis of SHMT2-associated genes indicated that they were enriched in energy metabolism and cell cycle-related pathways. These data suggest that SHMT2 might play an important role in cell proliferation by affecting mitochondrial function. In addition, previous reports have indicated that SHMT2 expression is upregulated in various types of cancer, including hepatocellular carcinoma (Ji et al., 2019), intrahepatic cholangiocarcinoma (Ning et al., 2018), breast cancer (Bernhardt et al., 2017) and gastrointestinal tumors (Liu et al., 2019). SHMT2 may be an oncogene (Lee et al., 2014), the succinylation of SHMT2 at K280 sites inhibits colorectal cancer cell growth (Yang et al., 2018); however, so far, no relevant literature on the association of SHMT2 with BLCA has been reported.

In the present study, we explored the prognostic value of SHMT2 by analyzing BLCA tissues. Next, we performed integrated bioinformatic analyses to investigate the potential role of SHMT2 in BLCA. In our current work, gene enrichment analysis showed that genes co-expressed with SHMT2 in BLCA are mainly involved in the cell cycle-related pathway. Finally, we analyzed the association between SHMT2 expression and cell survival and growth using SHMT2-silenced and SHMT2-overexpressed BLCA cell lines.

## Materials and Methods

### SHMT2 Expression in Human Cancers

SHMT2 mRNA expression in all available normal and tumor tissues were analyzed using the pan-cancer analysis from TNMplot database^1^. The TNMplot database includes 56,938 high quality samples, which was a new setup of gene expression omnibus (GEO), the Cancer Genome Atlas (TCGA), GTEx, and TARGET databases.

### SHMT2 Expression in Bladder Cancer

We first analyzed the SHMT2 differentially expression between BLCA and normal tissues using the Oncomine^2^ (Rhodes et al., 2004) database. To further verify SHMT2 mRNA expression in BLCA, BLCA and adjacent non-tumor tissue gene expression profiles were downloaded from TCGA^3^ (Oct, 2020), GEO [GSE3167 (Dyrskjøt et al., 2004) and GSE7476 (Mengual et al., 2009)], and TNMplot databases. Data were processed using R (v3.6.1) and Perl software, and the differentially expressed genes (DEGs) between BLCA and matched normal tissues were subsequently identified using the limma package in R software. DEGs were defined as differentially expressed if | log_2_FoldChange| > 1, and if the adjusted *p*-value (adj. *P*) < 0.05 for the microarray data.

### Survival and Clinical Pathology Correlation Analysis

The prognostic significance of SHMT2 mRNA expression levels was determined using the gene expression profiling interactive analysis (GEPIA) database^4^ (Tang et al., 2017) which was based on the TCGA-BLCA data, the log-rank test was used to compare survival curves in BLCA. And the correlation between SHMT2 expression and survival in BLCA was confirmed using the PrognoScan^5^ (Mizuno et al., 2009) database which was based on the GEO data, the threshold was set as a *P*-value < 0.05. The patients were divided into high and low SHMT2 expression groups according to the median expression of SHMT2. Next, correlation analyses between SHMT2 mRNA levels and different clinicopathological characteristics of BLCA patients were analyzed based on TCGA-BLCA clinical data.

### Enrichment Analysis of Genes Co-expressed With SHMT2

We first used co-expression analysis to identify the genes co-expressed with the SHMT2 in Lee CellLine2 based on the Oncomine database, and the genes strong correlated with SHMT2 (Pearson correlation coefficient > 0.6) were selected for the further function analysis. Subsequently, we used the search tool for the retrieval of interacting genes/proteins (STRING) website^6^ to query the protein (“SHMT2”) and organism (“Homo sapiens”). The main parameters were set as following: the score of minimum required interaction is 0.150, the number of interactors is no more than 50, and the network edges mean evidence from experiments. Then, SHMT2-binding proteins were obtained. Subsequently, we combined the above two data sets to perform enrichment analysis using Metascape^7^ (Zhou et al., 2019) database. Metascape is a new online gene annotation and analysis resource, and the users can analyze the interested genes only in three steps. Finally, gene set enrichment analysis (GSEA) was carried out to explore the role of SHMT2 in BLCA. BLCA samples from TCGA were divided into two different groups (high and low) based on the expression levels of SHMT2, and the median expression value was considered the cut-off point. A FDR < 0.05 was regarded as the cut-off criterium.

### Specimen Collection

Fifteen BLCA and matched normal tissues were obtained from the Guizhou Provincial People’s Hospital in order to verify the high mRNA and protein SHMT2 expression in BLCA tissues. Prior patient consent was obtained, and this study was approved by the Institutional Research Ethics Committee of Guizhou Provincial People’s Hospital.

### Cell Lines and Cell Transfection

SV-HUC-1 (a normal bladder epithelial cell line) was cultured in DME/F12 (SH30023; Hyclone, United States) and BLCA cell lines (5637, SW780, and T24) were cultured in RPMI-1640 (SH30809; Hyclone, United States). All cell lines were gifted from the Key Laboratory of Hubei Province for Digestive System Disease (Wuhan, Hubei, China). All media were supplemented with 10% fetal bovine serum (FBS; 141215; Sijiqing, Hangzhou, China). All cells were incubated at 37°C and 5% CO_2_ in a humidified incubator. Cells grown at 50% confluence were transfected using pcDNA3.1-flag-SHMT2 overexpression plasmid (OE-SHMT2) or pcDNA3.1 control vector (Vector) and siRNA-SHMT2 or siRNA-Negative control (siRNA-NC) (Suzhou GenePharma Co., Ltd.; Shanghai, China) by using Lipofectamine 2000 (11668019; Invitrogen; Carlsbad, CA, United States) according to the manufacturer’s instructions. After 24 h, T24 cells were harvested for further experiments.

The siRNA sequences were as follows:

Si-h-SHMT2_1: forward, 5′-GGAGAGUUGUGGACUUUA UTT-3′; reverse, 3′-AUAAAGUCCACAACUCUCCTT-5′;

Si-h-SHMT2_2: forward, 5′-CUGGCCUCAUUGACUACA ATT-3′; reverse, 3′-UUGUAGUCAAUGAGGCCAGTT-5′;

Si-h-SHMT2_3: forward, 5′-CCCUGCAGGUUCUGAAGA ATT-3′; reverse, 3′-UUCUUCAGAACCUGCAGGGTT-5′.

### Real-Time PCR

Total RNA was extracted from cultured cells using TRIzol reagent (15596–026; Invitrogen, Carlsbad, CA, United States) following the manufacturer’s instructions, and reverse transcription and real-time quantitative PCR were performed as previously described (Yang et al., 2021). All results for gene expression were normalized to that of GAPDH. Relative quantification was performed using the 2^–ΔΔ^ ^*CT*^ method. The primers used for qRT-PCR were as follows: SHMT2 sense, 5′-GCA TGTCACCGTTCCTCCTT-3′; anti-sense, 5′-GGGCATCTTC ACGCTCTATTT-3′; GAPDH sense, 5′-CATCATCCCTGC CTCTACTGG-3′; and anti-sense, 5′-GTGGGTGTCGC TGTTGAAGTC-3′.

### Western Blotting

Total protein samples were extracted from tissues and cultured cells. Then, the samples were separated using 10–12% SDS-PAGE and transferred onto a polyvinylidene fluoride membrane. After blocking with 5% non-fat milk in Tri-buffered saline for 1.5 h, the membranes were incubated at 4°C overnight with target antibodies against the following proteins: anti-SHMT2 (1:1,000, 11099-1-AP; Proteintech, Wuhan, China), anti-GAPDH (1:10,000; 60004-1-Ig; Proteintech, Wuhan, China),anti-Cleaved-caspase 3 (1:1,000, #9661S; CST, United States), anti-Cyclin D1 (1:1,000, 60186-1-Ig; Proteintech, Wuhan, China), anti-Cyclin E1 (1:1,000, 11554-1-AP; Proteintech, Wuhan, China), anti-Bcl-2 (1:1,000, #2872T; CST, United States), anti-Bax (1:1,000, 50599-2-Ig; Proteintech, Wuhan, China), anti-NF-κB P65 (1:1,000, #8242T; CST, United States), anti-p-NF-κB P65 (Ser536) (1:1,000, #3033; CST, United States), anti-STAT3 (1:1,000, #9139; CST, United States), anti-p-STAT3 (Tyr705) (1:2000, #9145; CST, United States). GAPDH was used as an internal loading control. After washing three times with TBST, the membranes were incubated with species-special secondary antibodies at room temperature for 1 h. Next, the membranes were detected by the ChemiDoc^TM^ Touch Imaging System (Bio-Rad, Hercules, United States). Finally, relative protein expression levels were assessed using the image J software.

### Cell Proliferation Assay

For cell proliferation analysis, T24 cells were seeded on a 96-well plate (3,000 cells/well) and transfected with siRNA-SHMT2_2, siRNA-SHMT2_3, or an siRNA negative control (NC), and 5637 cells were transfected with OE-SHMT2 or Vector. Cell Counting Kit-8 assay (CCK-8, BS350A; Biosharp, Shanghai, China) was used to analyze the cell growth at 0, 24, 48, and 72 h. Then, CCK-8 reagent was added to each well (10 μL/well) and cultured for 1 h at 37°C, and the optical density was measured at 450 nm.

### Clone Formation Assay

Transfected T24 cells and 5637 cells (siRNA-SHMT2_2, siRNA-SHMT2_3, siRNA NC; OE-SHMT2, Vector) were seeded onto six-well plates, with 200 cells per well and incubated for 14 days. Then, the cells were fixed with 10% formaldehyde for 15 min, stained with 4% crystal violet for 5 min, and counted. ImageJ (ImageJ 1.52a, United States) was used to calculate the number of colonies per well.

### Cell Cycle Analysis

A total of 48 h after cells transfection, cells were collected *via* centrifugation (×300 g) and resuspended in 100 μl ice-cold PBS. Cells were fixed with 70% ethanol (300 μl) at 4°C overnight. Then, 24 h later, cells were washed with 1 × PBS, resuspended in 1 ml of PBS and adjust the number of cells to 1 × 10^6^/ml. Subsequently, we added 10 μl RNAse A (CY2001-O; Tianjin Sungene Biotech Co., Ltd., Tianjin, China) and 10 μl propidium iodide (AO2001-02P-G; Tianjin Sungene) into 500 μl dyeing buffer for one sample analysis. After incubated with dyeing buffer for 30 min at 37°C, cell samples were analyzed using flow cytometry (FACSCalibur, BD Biosciences, San Jose, CA, United States).

### Apoptosis Analysis

After transfection with 48 h, cells were collected and centrifuged (×300 *g*, 5 min), the supernatants were discarded and resuspended in PBS for twice, and then resuspended in 300 μl binding buffer. Next, cells were stained with 5 μl annexin V/FITC for 10 min, and 5 μl PI (Tianjin Sungene) for another 5 min in the dark, according to the manufacturer’s instructions. Finally, the samples were analyzed using flow cytometry (BD Biosciences).

### Statistical Analysis

R (version 3.6.1), SPSS Statistics 25.0 (IBM, Inc., Chicago), and GraphPad Prism 7 (GraphPad Software, CA, United States) software were used to perform all statistical analyses. To compare survival curves, we used the log rank test to calculate the HR and logrank *P*-value in Kaplan-Meier Plotter and GEPIA. A univariate Cox regression model was used to calculate the HR and Cox *P* value in PrognoScan. Differences in quantitative data between the two groups were analyzed using paired or unpaired Student’s *t*-tests, Mann-Whitney U-tests, or Dunnett’s *t*-tests as appropriate; a two-way ANOVA with Sidak’s multiple comparisons was applied to multiple comparisons. We considered a *P* < 0.05 as statistically significant (^∗^*P* < 0.05, ^∗∗^*P* < 0.01, ^∗∗∗^*P* < 0.001, and ^****^P < 0.0001).

## Results

### SHMT2 Expression in Pan-Cancer

**FIGURE 1:**
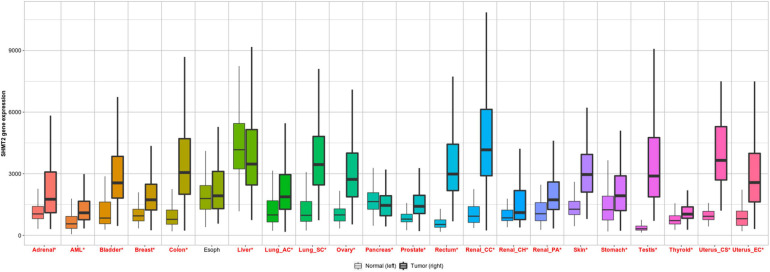
SHMT2 expression in pan-cancer. Differential SHMT2 expression analysis in human tumors and normal tissues in TNMplot database. Significant differences are marked with red *P < 0.05 was recognized as statistically significant.

To investigate the differential expression of SHMT2 between human tumors and normal tissues, SHMT2 expression levels were analyzed using the TNMplot database. It was showed that compared to the respective normal tissues, SHMT2 was highly expressed in adrenal cancer, bladder cancer, breast cancer, liver cancer, colorectal cancer, lung cancer, ovary cancer, pancreatic cancer, prostatic cancer, stomach cancer, testis cancer, skin cancer, thyroid cancer, uterus cancer, renal cancer as well as acute myelogenous leukemia (AML). In addition, SHMT2 was also upregulated in esophageal cancers, but there was no statistical significance (Figure 1).

### SHMT2 Overexpression Was Frequently Identified in Bladder Cancer

**FIGURE 2:**
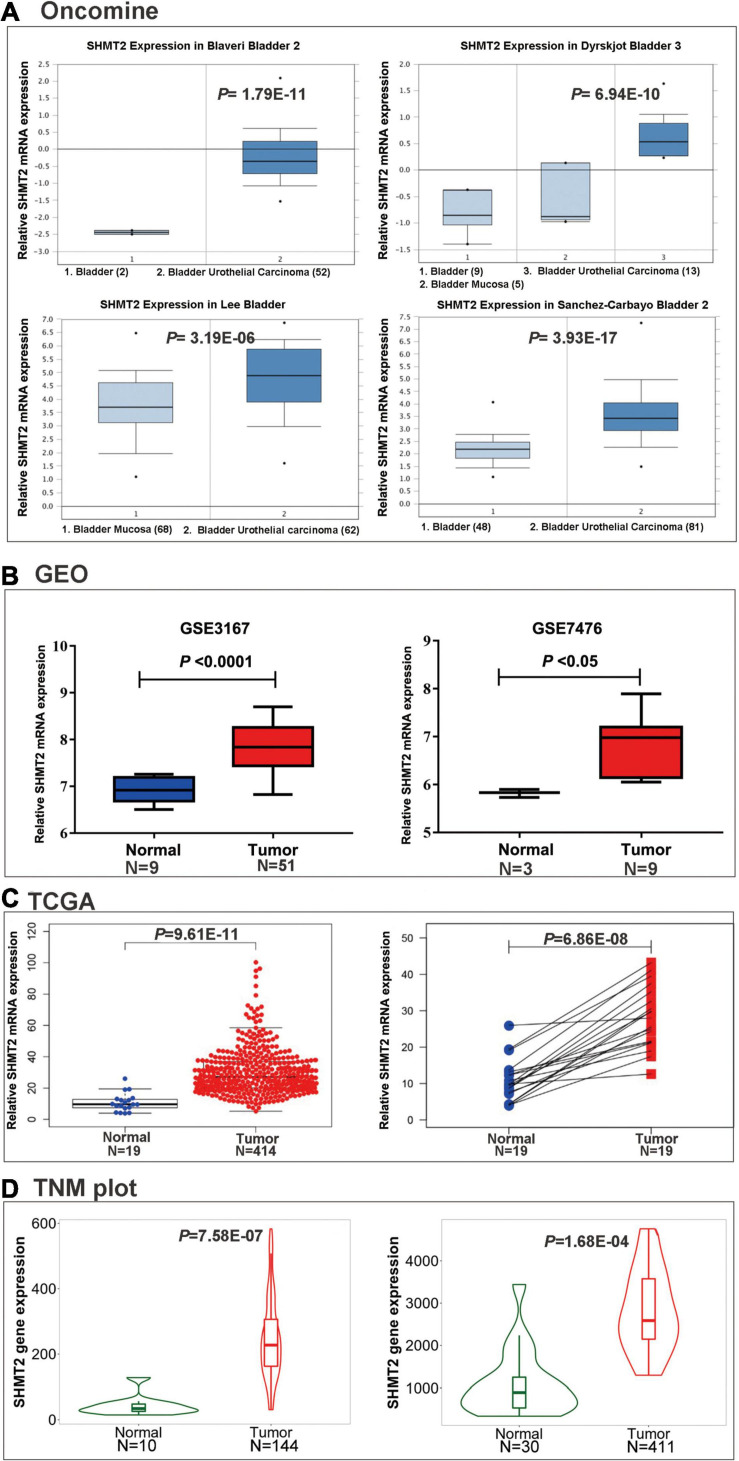
SHMT2 expression in BLCA tissues. **(A)** SHMT2 was overexpressed in BLCA tissues than normal tissues in four oncomine data sets (Blaveri bladder, *P* = 1.79E-11; Dyrskjot bladder, *P* = 6.94E-10; Lee bladder, *P* = 3.19E-06; Sanchez-Carbayo bladder, *P* = 3.93E-17). **(B)** SHMT2 was upregulated in BLCA tissues in two GEO data sets (GSE3167, *P* < 0.0001; GSE7476, *P* < 0.05). **(C)** SHMT2 expression in TCGA-BLCA cases was significantly higher than that in normal bladder tissues (*P* < 0.0001). **(D)** SHMT2 was overexpressed in BLCA tissues than normal tissues in TNMplot database (left-Genechip, *P* = 7.58E-07; right-RNA-seq, *P* = 1.68E-04). *P* < 0.05 was recognized as statistically significant.

To explore SHMT2 expression in BLCA, we first analyzed the data from the Oncomine database. As expect, SHMT2 were highly upregulated in the four bladder carcinoma studies: Blaveri bladder, *P* = 1.79E-11; Dyrskjot bladder, *P* = 6.94E-10; Lee bladder, *P* = 3.19E-06; Sanchez-Carbayo bladder, *P* = 3.93E-17 (Dyrskjot et al., 2004; Blaveri et al., 2005; Sanchez-Carbayo et al., 2006; Lee et al., 2010; Figure 2A). Next, GSE3167 (nine normal bladder samples versus 51 BLCA samples, *P* < 0.0001), GSE7476 (three normal bladder samples versus nine BLCA samples, *P* < 0.05) (Figure 2B), and TCGA RNA-Seq expression data (19 normal bladder tissues versus 414 BLCA tissues, *P* = 9.61E-11; 19 BLCA and 19 matched normal bladder tissues, *P* = 6.86E-08) were selected to verify that SHMT2 was overexpressed in BLCA tissues (Figure 2C). Additionally, in order to further determine SHMT2 mRNA expression in BLCA, the analysis was expanded to a larger cohort of TNMplot to confirm our observations. As a result, SHMT2 expression was significantly upregulated both in BLCA-Genechip (10 normal bladder samples versus 144 BLCA samples, *P* = 7.58E-07) and RNA-seq (30 normal bladder samples versus 411 BLCA samples, *P* = 1.68E-04) compared to that in the normal tissues (Figure 2D).

### Overexpression of SHMT2 Was Related With a Poor Prognosis of BLCA Patients

**FIGURE 3:**
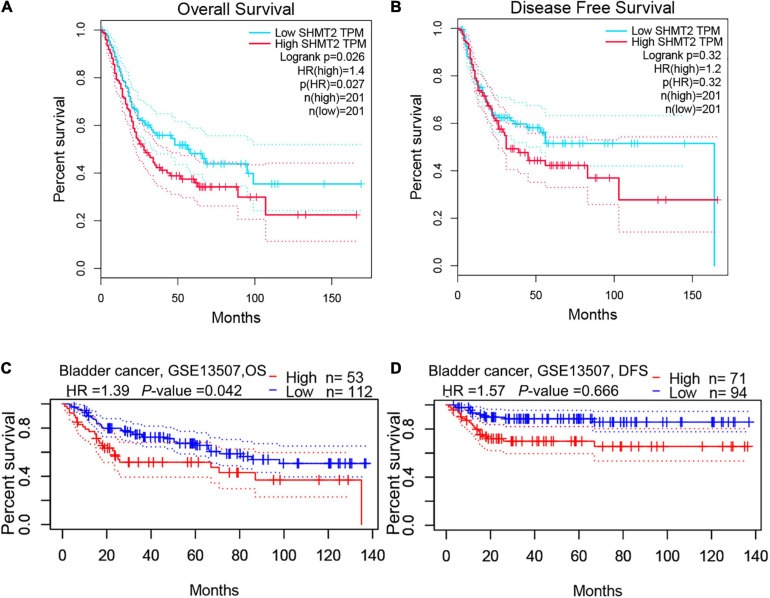
High SHMT2 expression predicts poorer prognosis in BLCA. Based on GEPIA database, we found SHMT2 overexpression was correlated with poorer OS (*P* = 0.026) **(A),** but showed no correlation with DFS (*P* = 0.32) **(B).** As expect, a high SHMT2 expression was significantly associated with a poorer OS (*P* = 0.042) of BLCA patients **(C)**, but was not correlated with the DFS (*P* = 0.066) based on GSE13507 in PrognoScan database **(D)**. overall survival, OS; disease-free survival, DFS, *P* < 0.05 was recognized as statistically significant.

**FIGURE 4:**
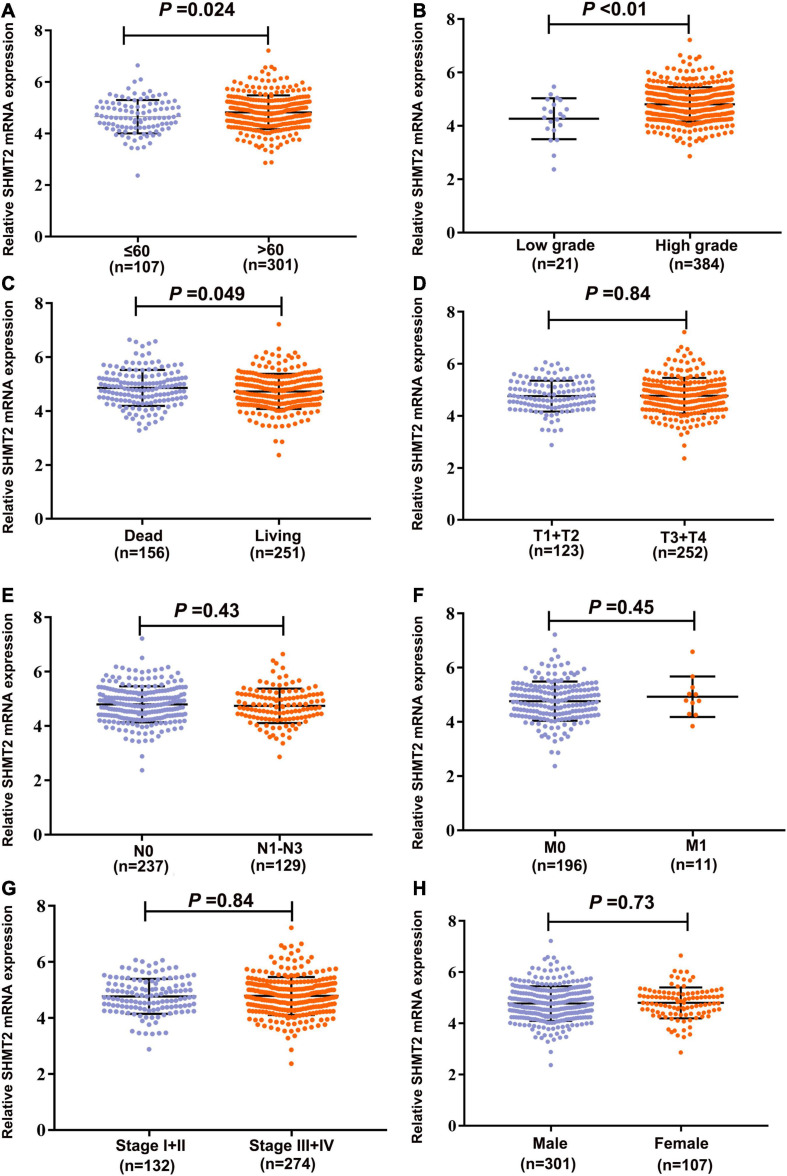
Correlation analyses between SHMT2 expression and different clinicopathological characteristics of BLCA patients. The association between SHMT2 expression and clinicopathological characteristics of BLCA patients, including **(A)** Age (*P* = 0.024); **(B)** Tumor grade (*P* < 0.001); **(C)** Overall survival (*P* = 0.049); **(D)** Tumor (T) stage (*P* = 0.84); **(E)** Node (N) stage (*P* = 0.43); **(F)** Metastasis (M) stage (*P* = 0.45); **(G)** Tumor node metastasis (TNM) stage (*P* = 0.84); **(H)** Gender (*P* = 0.73). *P* < 0.05 was recognized as statistically significant.

To evaluate the association between SHMT2 expression and the prognosis of BLCA patients, we used the data from the GEPIA databases (*N* = 402) to perform the Kaplan–Meier survival curve analysis. It was indicated that BLCA patients with a high SHMT2 mRNA expression were correlated with a poorer overall survival (OS) [Hazard Ratio (HR) = 1.4, *P*(HR) = 0.027] than patients with a low SHMT2 mRNA expression (Figure 3A); however, a high SHMT2 expression was not related to disease-free survival (DFS) (HR = 1.2, *P*(HR) = 0.32 (Figure 3B). We then demonstrated the prognostic value of SHMT2 by analyzing BLCA patients using GEO. Similar to the findings obtained using GEPIA, a high SHMT2 expression was significantly associated with a poorer OS of BLCA patients [GSE13507 (Kim et al., 2010), total number = 165, HR = 1.39, *P* = 0.042], but was not correlated with the DFS (total number = 165, HR = 1.57, *P* = 0.067) of BLCA patients (Figures 3C,D). Next, the association between SHMT2 expression and clinicopathological features was analyzed in 408 TCGA-BLCA patients (Supplementary Table 1). The results showed that SHMT2 expression was significantly higher in patients older than 60 years (*P* = 0.024) (Figure 4A), with a high tumor grade (*P* = 0.0003 < 0.001) (Figure 4B), and in dead BLCA patients (*P* = 0.049) (Figure 4C), but showed no association with tumor (T) stage (*P* = 0.84), node (N) stage (*P* = 0.43), metastasis (M) stage (*P* = 0.45), TNM stage (*P* = 0.84) and gender (*P* = 0.73) (Figures 4D–H).

### Enrichment Analysis of Genes Co-expressed With SHMT2

**FIGURE 5:**
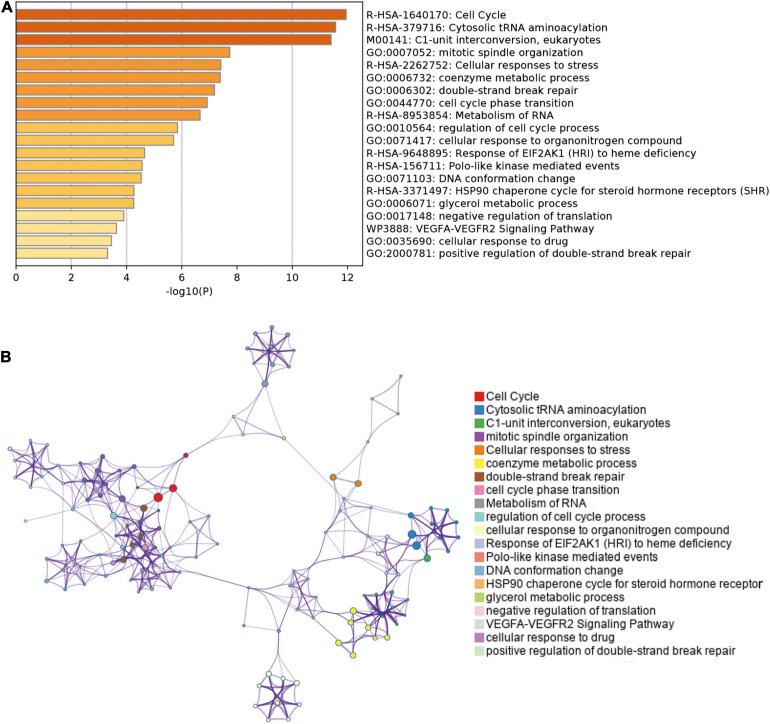
Enrichment analysis of genes co-expressed with SHMT2 in Metascape. **(A)** Top 20 enriched functional terms of SHMT2-related genes are shown in the bar graph, terms are colored according to *P*-values. **(B)** Network of enriched functional terms, nodes shown in the same color share one term. *P* < 0.05 was recognized as statistically significant.

**FIGURE 6:**
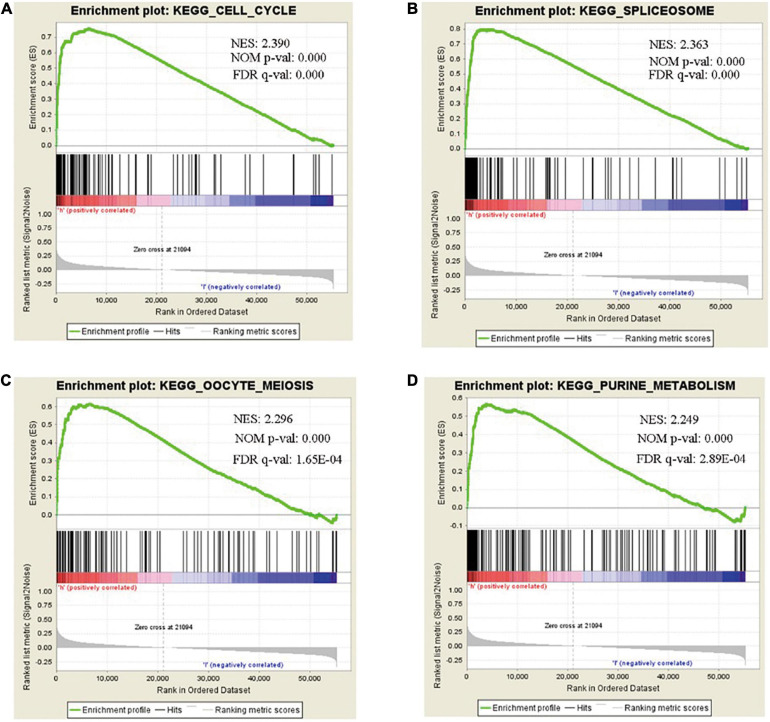
Gene set enrichment analysis (GSEA) of SHMT2 in BLCA. GSEA analysis showed that SHMT2 may regulate the **(A)** cell cycle (NES = 2.390, NOM-*P* < 0.0001), **(B)** spliceosome (NES = 2.363, NOM-*P* < 0.0001), **(C)** oocyte meiosis (NES = 2.296, NOM-*P* < 0.0001), and **(D)** purine metabolism (NES = 2.249, NOM-*P* < 0.0001) pathways in BLCA development. *P*-value < 0.05 was recognized as statistically significant.

We analyzed and extracted the genes strong correlated with SHMT2 from the Oncomine database (Lee CellLine2, co-expression coefficient > 0.6): YARS (co-expression coefficient:0.758), MARS (co-expression coefficient:0.758), RBCK1(co-expression coefficient:0.758), TRIB3 (co-expression coefficient:0.758), SLC38A1 (co-expression coefficient:0.739), ATF4 (co-expression coefficient:0.730), CCNB1IP1 (co-expression coefficient:0.664), EIF2S2 (co-expression coefficient:0.647), EPRS (co-expression coefficient:0.628), SARS (co-expression coefficient:0.628), GARS (co-expression coefficient:0.628), CARS (co-expression coefficient:0.628), PHGDH (co-expression coefficient:0.628), PCK2 (co-expression coefficient:0.758) and PSAT1 (co-expression coefficient:0.628) (Supplementary Table 2 and Supplementary Figure 1). Next, the proteins interacted with SHMT2 were constructed in a network (Supplementary Figure 2). Furthermore, the genes from the above two data sets were for enrichment analysis. Then, Metascape database was used to determine the biological functions of SHMT2 and its co-expressing genes, including their molecular functions, and biological pathways. The genes associated with biological pathways were mainly enriched in cell cycle associated pathway, cytosolic tRNA aminoacylation, cellular responses to stress, coenzyme metabolic process, double-strand break repair, metabolism of RNA (Figures 5A,B). To identify the signaling pathways activated by the differential upregulation of SHMT2 expression in bladder cancer, GSEA analysis of the low and high SHMT2 expression datasets was performed. Finally, the results also indicated that the cell cycle-related pathways [normalized enrichment score (NES) = 2.390, nominal (NOM)-*P* < 0.0001, false discover rate (FDR)-q < 0.0001], spliceosome (NES = 2.363, NOM-*P* < 0.0001, FDR-q < 0.0001), oocyte meiosis (NES = 2.296, NOM-*P* < 0.0001, FDR-q = 1.65E-04), and purine metabolism (NES = 2.249, NOM-*P* < 0.0001, FDR-q = 2.89E-04) were significantly enriched in high SHMT2 expression dataset (Supplementary Table 3 and Figures 6A–D). These results reveal that SHMT2 might involve in the occurrence and progression of BLCA by regulating cell cycle-related pathways.

### Silencing of SHMT2 Inhibits Cell Proliferation, Overexpression of SHMT2 Promotes Cell Proliferation

**FIGURE 7:**
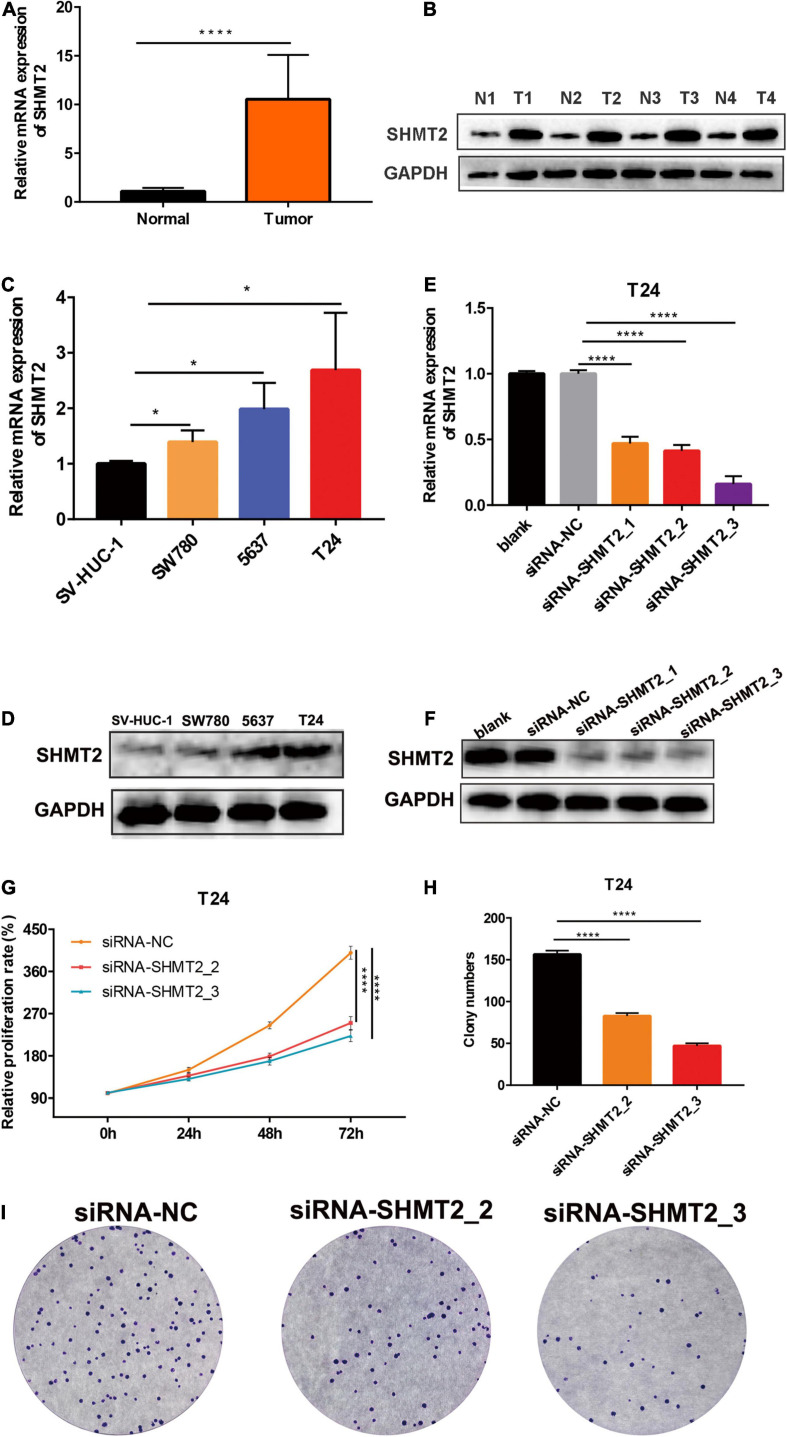
Knockdown of SHMT2 inhibited bladder cancer cell proliferation. **(A)** SHMT2 mRNA expression level was higher in bladder cancer tissues than that in normal tissues (****P < 0.0001). **(B)** Representative western blotting images showed that SHMT2 protein expression level was higher in bladder cancer tissues. **(C)** SHMT2 mRNA expression level was higher in BLCA cell lines (SW780, 5637, and T24) than that in bladder normal cell line SV-HUC-1 (*P < 0.05). **(D)** SHMT2 protein expression level was higher in BLCA cells. **(E,F)** SHMT2 mRNA and protein expression levels were examined in SHMT2-silenced T24 cells (****P < 0.0001). **(G)** Knockdown of SHMT2 decreased the BLCA cell proliferation rates using CCK-8 assay. (*n* = 3; ****P < 0.0001). **(H,I)** Knockdown of SHMT2 reduced the colony numbers, graph illustrating quantified values (*n* = 3; ****P < 0.0001). CCK-8, cell counting kit-8, *P*-value < 0.05 was recognized as statistically significant.

**FIGURE 8:**
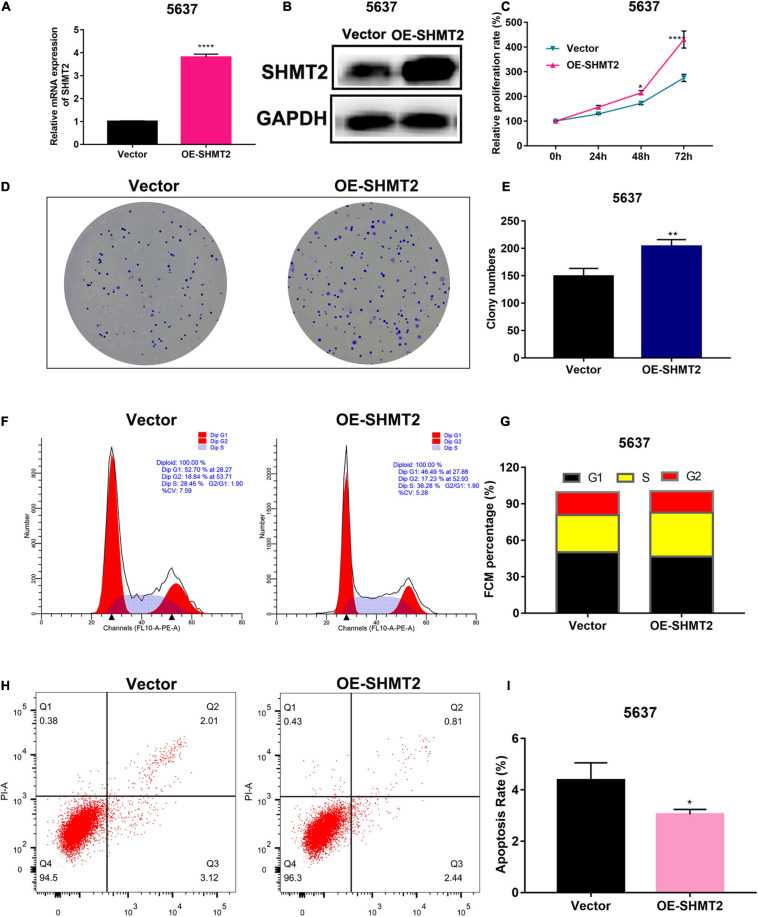
Overexpression of SHMT2 promoted bladder cancer cell proliferation. **(A,B)** SHMT2 mRNA and protein expression levels were examined in SHMT2-overexpressed 5637 cells (****P < 0.0001). **(C)** Overexpression of SHMT2 increased the BLCA cell proliferation rates using CCK-8 assay. (*n* = 3, *P < 0.05, ****P < 0.0001). **(D,E)** Overexpression of SHMT2 increased the colony numbers, graph illustrating quantified values (*n* = 3; **P < 0.01). **(F,G)** Overexpression of SHMT2 promoted cell cycle progression, distribution of cells in three cell cycle phases was examined by flow cytometry assay, and the graph shows quantification for each phase. **(H,I)** Overexpression of SHMT2 inhibited the rate of apoptosis, for measurement of apoptotic cells, cells were stained with both AV and PI, and analyzed by an image flow assay, graph illustrating the percentage of apoptotic cells (*n* = 3; *P < 0.5). AV, annexin V; PI, propidium iodide, OE, overexpression, *P*-value < 0.05 was recognized as statistically significant.

To determine whether SHMT2 could be a therapeutic target in BLCA, we first detected the mRNA and protein expressions of SHMT2 in BLCA tissues. It was indicated that SHMT2 was highly expressed in BLCA tissues compared to normal tissues (Figures 7A,B). Then, we tested SHMT2 expression in BLCA cell lines (SW780, 5637, and T24). It was shown that SHMT2 was significantly upregulated in SW780 (*P* = 0.0363), 5637 (*P* = 0.0471) and T24 cells (*P* = 0.0237), compared to normal bladder uroepithelial cells (Figures 7C,D). Therefore, the T24 cell line was selected for further loss-of-function analysis, 5637 cell line was for gain-of-function analysis. Next, SHMT2 was inactivated using siRNA in T24 cells (Figures 7E,F) to investigate its underlying functions in BLCA. Based on the results of qPCR and western blotting, the silencing efficiency of siRNA-SHMT2_2 and siRNA SHMT2_3 was more than 70%. Therefore, siRNA-SHMT2_2 and siRNA SHMT2_3 were chose to further analysis. We found that knockdown of SHMT2 can significantly inhibit cell growth [siRNA-SHMT2_2 group, 48 h (178.84 ± 7.01%),*P* < 0.0001; siRNA-SHMT2_3 group, 48 h (168.71 ± 8.28%),*P* = 0.0387 versus siRNA-negative control (NC) group (245.28 ± 7.3%); siRNA-SHMT2_2 group 72 h (250.03 ± 13.81%),*P* < 0.0001;siRNA-SHMT2_3 group 72 h (222.64 ± 12.27%),*P* < 0.0001 versus siRNA-NC group (399.89 ± 13.87%)] in T24 cells (Figure 7G). And, knockdown of SHMT2 can significantly decrease the colony number [siRNA-SHMT2_2 group (82.67 ± 3.51), *P* < 0.0001; siRNA-SHMT2_3 group (47 ± 3), *P* < 0.0001 versus siRNA-NC group (156.33 ± 4.51)] in T24 cells (Figures 7H,I). Additionally, the 5637 cells transfected with SHMT2 overexpression plasmid was detected using qPCR and western blotting (Figures 8A,B). And we also found that upregulation of SHMT2 promoted cell proliferation [OE-SHMT2 group, 48 h (171.08 ± 5.83%) versus vector group (211.14 ± 8.48%), *P* = 0.0261; OE-SHMT2 group, 72 h (275.49 ± 14.47%) versus vector group (412.80 ± 34.54%), *P* < 0.0001] using CCK8 assay (Figure 8C). Furthermore, overexpression of SHMT2 can significantly increase the colony number [OE-SHMT2 group, (207 ± 12%) versus vector group (145 ± 14%), *P* = 0.0073] (Figures 8D,E). These results suggest that silencing of SHMT2 inhibited cell proliferation, overexpression of SHMT2 promoted cell proliferation.

### SHMT2 Regulates Cell Cycle Progression and Apoptosis

**FIGURE 9:**
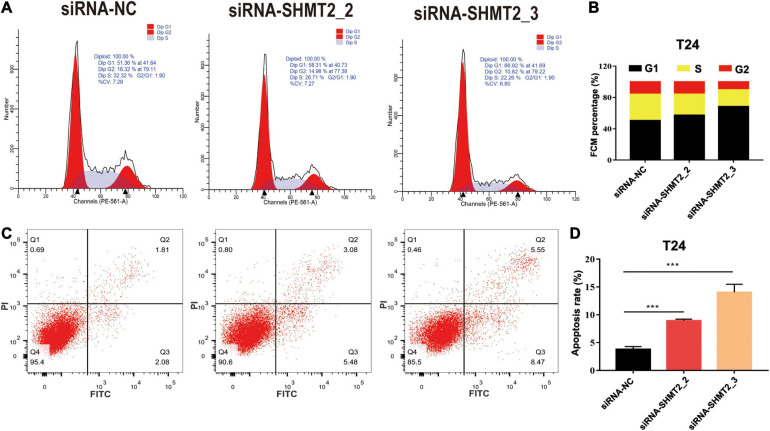
Knockdown of SHMT2 impaired cell cycle progression and induced apoptosis. Knockdown of SHMT2 impaired cell cycle progression, **(A)** distribution of cells in three cell cycle phases was examined by flow cytometry assay, and the graph shows quantification for each phase **(B)** (*n* = 3; ***P < 0.001). Knockdown of SHMT2 increased the rate of apoptosis, for measurement of apoptotic cells, cells were stained with both AV and PI, and analyzed by an image flow assay **(C)**, **(D)** graph illustrating the percentage of apoptotic cells (*n* = 3; ***P < 0.001). AV, annexin V; PI, propidium iodide, *P*-value < 0.05 was recognized as statistically significant.

Above enrichment analysis has indicated that SHMT2 might regulate BLCA cell cycle progression. To explore how SHMT2 affects BLCA cell growth, the cell cycle were analyzed using flow cytometry and western blotting. We found that overexpression of SHMT2 decreased the percentage of cells in the G1 phase [OE-SHMT2 group (46.49 ± 1.8%), versus vector group (50.12 ± 1.78%), *P* < 0.01] and increased the percentage of cells in the S phase [OE-SHMT2 group (36.28 ± 6.03%), versus vector group (30.59 ± 1.61%)] (Figures 8F,G). In addition, silencing of SHMT2 increased the percentage of cells in the G1 phase [siRNA-SHMT2_2 group (57.24 ± 1%), *P* = 0.0016; siRNA-SHMT2_3 group (68.26 ± 1.25%), *P* < 0.0001 versus siRNA-NC group (50.59 ± 1.14%)] and decreased the percentage of cells in the S phase [siRNA-SHMT2_2 group (27.01 ± 0.72%), *P* = 0.008; siRNA-SHMT2_3 group (21.63 ± 0.98%), *P* = 0.001 versus siRNA-NC group (33.47 ± 2.16%)] (Figures 9A,B).

Cell proliferation and apoptosis are two important cell processes which to balance cell growth. Our above results had showed that knockdown of SHMT2 suppressed cell proliferation, so we further analyzed the effects of SHMT2 in apoptosis. The results suggested that the proportion of apoptotic cells significantly decreased in SHMT2 overexpressed 5637 cells [OE-SHMT2 group (3.01 ± 0.2%), versus vector group (4.18 ± 0.67%), *P* = 0.0297] (Figures 8H,I), but increased in SHMT2-silenced T24 cells [siRNA-SHMT2_2 group (8.92 ± 0.31%), *P* = 0.0001; siRNA-SHMT2_3 group, *P* = 0.0003 (14.05 ± 1.45%) versus siRNA-NC group (3.79 ± 0.51%)] (Figures 9C,D). These data indicate that knockdown of SHMT2 could promote BLCA apoptosis, while overexpression of SHMT2 could inhibit apoptosis.

**FIGURE 10:**
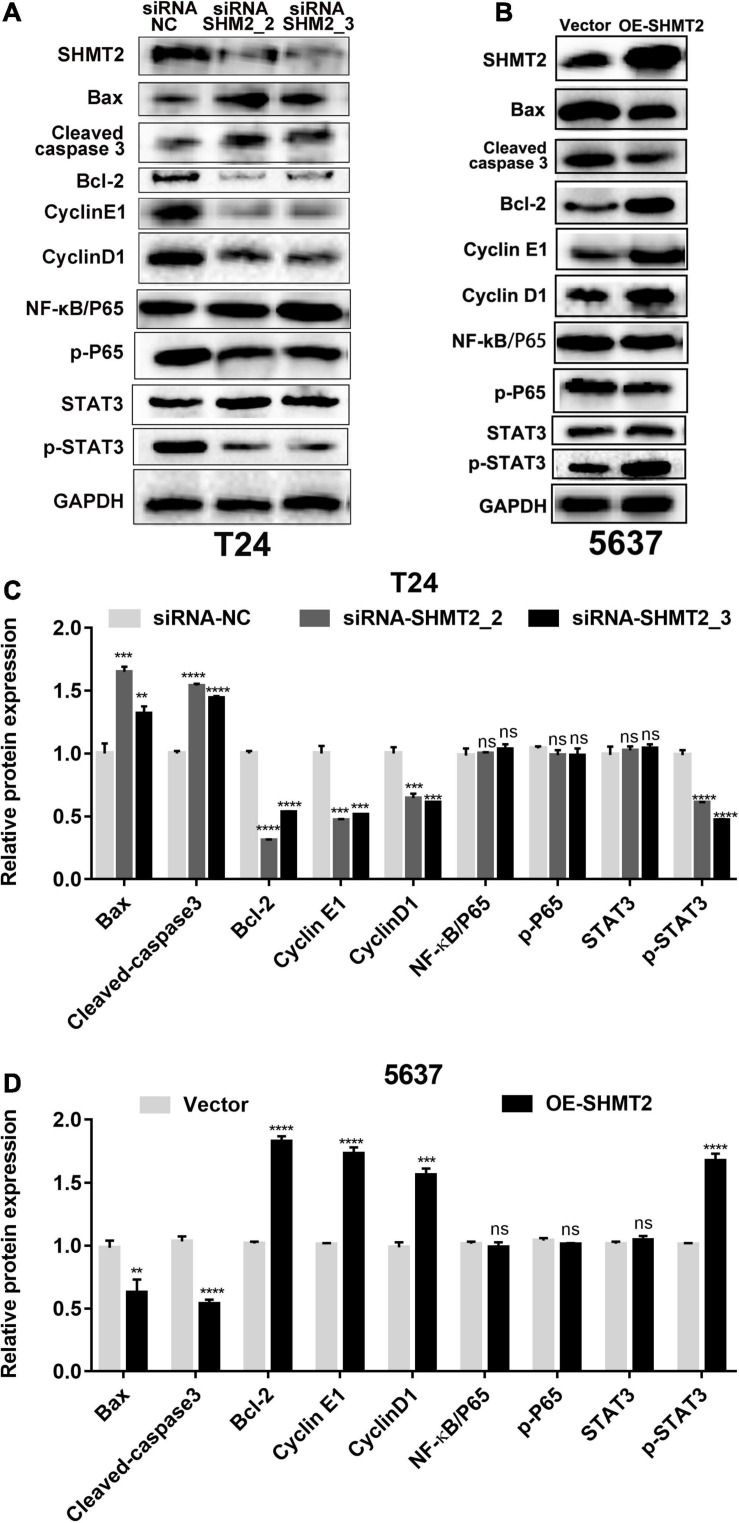
Protein expression analysis of downstream of SHMT2. **(A–D)** Knockdown of SHMT2 increased the apoptotic protein expression levels of Bax and cleaved-caspase 3, but decreased the antiapoptotic protein expression levels of Bcl-2; overexpression of SHMT2 decreased the protein expression of Bax and cleaved-caspase 3, but increased the protein expression of Bcl-2. Knockdown of SHMT2 reduced the cell cycle-related protein expression levels of cyclin E1 and cyclin D1, overexpression of SHMT2 elevated the protein expression of cyclin E1 and cyclin D1. SHMT2 did not affect the expression of NF-κB/P65, p-P65 and STAT3, knockdown of SHMT2 reduced the protein expression levels of p-STAT3, overexpression of SHMT2 elevated the protein expression levels of p-STAT3 (*n* = 3; ***P < 0.001, ****P < 0.0001). *P*-value < 0.05 was recognized as statistically significant.

Furthermore, western blotting assay was used to explore the mechanism how SHMT2 affected BLCA cells proliferation and apoptosis. The results showed that silencing of SHMT2 can decrease the expression levels of G1/S transition related protein (cyclin E1 and cyclin D1), while upregulation of SHMT2 can increase the expression levels of cyclin E1 and cyclin D1, indicating that SHMT2 facilitates the G1-to-S phase transition in BLCA cells. In addition, we also found that knockdown of SHMT2 can elevate the protein expression level of Bax and cleaved-caspase 3, but reduce the protein expression level of Bcl-2. While overexpression of SHMT2 can decrease the protein expression level of Bax and cleaved-caspase 3, but increased the protein expression level of Bcl-2. NF-κB signaling plays an essential role in cell growth, differentiation and apoptosis, and STAT3 is a well-known oncoprotein and a hub regulator of numerous signaling pathways. Hence, we further detected the protein expression levels of NF-κB and STAT3 in SHMT2-silenced T24 cells and SHMT2-overexpressed 5637 cells, respectively. As a result, we found that SHMT2 did not affect the expression levels of NF-κB/P65 (including total protein expression level and phosphorylated protein expression level of NF-κB/P65) and the total protein expression levels of STAT3. However, knockdown of SHMT2 decreased the phosphorylated protein expression level of STAT3 in T24 cells, overexpression of SHMT2 increased the phosphorylated protein expression level of STAT3 in 5637 cells (Figures 10A–D). Therefore, our current study revealed that SHMT2 promoted BLCA cells growth through regulating STAT3 signaling pathway.

## Discussion

Despite the recent advances in the surgical and medical treatment of BLCA, mortality remains high. Understanding the etiology and mechanism of BLCA progression is crucial to improve the survival rate and prevent the occurrence of BLCA. Recently, the rapid development of microarray technology has been widely used in gene expression level comparison, disease progression prediction, and improvement of diagnosis and prognostic assessment.

SHMT2 is a key metabolic enzyme that is involved in folate-mediated one-carbon metabolism and in serine-to-glycine reversible conversion. SHMT2 is mainly localized in the mitochondria and is also found in the cytoplasm and nucleus (Zheng et al., 2013). Mitochondria, which have been recognized as a central hub in coordinating cellular stress responses, mediate multiple biological cellular processes, including proliferation and apoptosis (Ahn and Metallo, 2015), while a proper mitochondrial translation initiation requires serine catabolism by SHMT2 (Minton et al., 2018). Researchers have found that mitochondrial respiration defects in mice deficient in the SHMT2 gene were embryonic lethal (Tani et al., 2018). Similarly, cancer cells need to adjust their metabolic processes to support a rapid proliferation. SHMT2 has been identified as an oncogene (Lee et al., 2014), and desuccinylation of SHMT2 is a pivotal cancer cell signal to adjust the serine metabolic process to ensure a rapid development (Yang et al., 2018). Additionally, SHMT2 can drive glioma cell survival in ischemia (Kim et al., 2015), which showed that during the process of cancer cell adaptation to the tumor environment, SHMT2 is required. Previous studies reported that SHMT2 can promote liver regeneration through the glycine-activated Akt/mTOR pathway (Wang et al., 2019), while a downregulated SHMT2 can suppress tumorigenesis in human hepatocellular carcinoma (Woo et al., 2016). Moreover, SHMT2 has been found to be associated with a poor prognosis in hepatocellular carcinoma (Ji et al., 2019), intrahepatic cholangiocarcinoma (Ning et al., 2018), breast cancer (Bernhardt et al., 2017) and gastrointestinal tumors (Liu et al., 2019). In addition, ERRα can activate SHMT2 transcription by targeting its promoter region to enhance breast cancer resistance to lapatinib (Li et al., 2020).

To date, SHMT2 has been reported to be overexpressed in malignant lesions; however, there is no related research in bladder urothelial carcinoma, which was done in this paper for the first time. Therefore, the results of this study have clinical significance for BLCA. First, we found that SHMT2 was highly expressed in all most tumor tissues base on the TNMplot database. Next, we investigated that SHMT2 was upregulated in BLCA tissues using Oncomine, TCGA, GEO, and TNMplot datasets. Then, we used the GEPIA and PrognoScan databases to reveal that high SHMT2 expression was associated with a poor overall prognosis. These data reveal that SHMT2 is a prognostic and diagnostic indicator in BLCA patients. Moreover, TCGA-BLCA clinical data were used to determine the correlation between the expression of SHMT2 and clinicopathological parameters. It was indicated that the elderly and high-grade BLCA patients might be associated with a SHMT2 high expression. However, sex and TNM stage were not related to SHMT2 expression. To our knowledge, the signaling pathways and their exact biological roles in BLCA remain unclear. To predict the role of SHMT2 in BLCA, molecular function and pathway enrichment analysis for SHMT2 and its co-expressing genes were explored. As a result, these genes were mainly participate in the regulation of cell cycle process, which are important for cell growth and survival. Additionally, previous reports have shown that most of these genes that are co-expressed with SHMT2, including EIF2S2 (Zhang J. et al., 2020), PCK2 (Chen et al., 2020), PHGDH (Yoshino et al., 2020), PSAT1 (Chan et al., 2020), RBCK1 (Yu et al., 2019), TRIB3 (Lin et al., 2018) and YARS (Zhang C. et al., 2020), play an oncogenic role in tumorigenesis and progression, which further revealed that SHMT2 might also play an important role in BLCA. Moreover, GSEA suggested that SHMT2 might be involved in BLCA formation through cell cycle-associated signaling pathways.

To determine the role of SHMT2 in BLCA, we verified that both SHMT2 mRNA and protein expression levels were overexpressed in BLCA tissues and cell lines (SW780, 5637, and T24). Thus, T24 cells with relative high SHMT2 expression were selected for loss-of-function experiments. Next, we verified three small interfering RNA of SHMT2 in T24 cells, and two siRNA-SHMT2 (siRNA-SHMT2_2 and siRNA-SHMT2_3) with high silencing efficiency were selected for further functional experiment. We first found that knockdown of SHMT2 (both in siRNA-SHMT2_2 and siRNA-SHMT2_3 groups) inhibited T24 cell survival and growth using CCK8 and clone formation assays, and knockdown of SHMT2 in siRNA-SHMT2_3-transfected T24 cells may more significantly inhibit cell growth than siRNA-SHMT2_2 group.

Since we found SHMT2 regulated BLCA cells proliferation, and cell cycle is an important process that affects cell proliferation, while G1 phase to S phase transition is a key step in cell cycle progression. Moreover, the GSEA analysis in this study predicts that SHMT2 might involve in regulating BLCA cell cycle. Therefore, we next analyzed the effects of SHMT2 in BLCA cell cycle, and the results demonstrated that overexpression of SHMT2 promote G1/S phase transition, silencing of SHMT2 significantly inhibited cell cycle G0/G1 phase to S phase transition by downregulating cyclin E1 and cyclin D1 protein expression. These findings suggest that SHMT2 could promote G1/S transition in BLCA cells.

Cell proliferation and apoptosis are two important cellular processes in tumorigenesis, the imbalance between the two processes may lead to abnormal development of the body and lead to tumor. And the suppression of cell proliferation prompted us to investigate the effects of SHMT2 silencing on apoptosis. The apoptosis of BLCA cells was evaluated using annexin V-FITC/PI flow cytometry. It was indicated that overexpression of SHMT2 decreased the rate of apoptotic in 5637 cells, knockdown of SHMT2 increased the rate of apoptotic cells in T24 cells by upregulating Bax and cleaved-caspase 3 expression. These findings suggest a vital role of SHMT2 in the growth and mitochondrial apoptosis of BLCA.

It is well known that NF-κB and STAT3 signaling plays an essential role in cell growth, differentiation, apoptosis, and previous study revealed that SHMT2 is a new player in STAT3 signaling in cancer (Marrocco et al., 2019). Therefore, we further analyzed the protein expression of SHMT2 potential downstream pathway. These results indicated that neither SHMT2 knockdown nor SHMT2 overexpression affected NF-κB/P65 (total protein and phosphorylation protein) protein expression. However, our results showed that silencing of SHMT2 decreased the phosphorylation protein expression of STAT3, upregulation of SHMT2 increased the phosphorylation protein expression of STAT3. Above all, this current study suggests that SHMT2 promoted BLCA cells growth and induced apoptosis mainly *via* enhancing the STAT3 phosphorylation expression at Tyr705 sites.

In summary, data from public databases and our samples allowed us to identify SHMT2 as a potential oncogene in BLCA. Our results indicated that SHMT2 was highly expressed in BLCA tissues and cells. Overexpression of SHMT2 promoted the BLCA cells growth, cell cycle progression and inhibited apoptosis, knockdown of SHMT2 suppressed the BLCA cells growth, impaired cell cycle progression, and promoted apoptosis through STAT3 signaling pathway.

However, there are some limitations to this study. First, the study cohort samples from our hospital are relative small, which is needed to enlarge. Second, future work will be needed to investigate the effect of SHMT2 *in vivo*.

## Conclusion

In conclusion, the present results revealed that SHMT2 is significantly upregulated in BLCA tissues and cell lines. A high SHMT2 expression is correlated with the tumor histological grade of BLCA and predicts a poorer overall survival. Overexpression of SHMT2 promote BLCA cell growth, knockdown of SHMT2 may suppress BLCA cell proliferation by impairing the cell cycle and inducing apoptosis. Therefore, SHMT2 could serve as a promising diagnostic and prognostic biomarker and a novel therapeutic target for BLCA.

## Data Availability Statement

The original contributions presented in the study are included in the article/Supplementary Material, further inquiries can be directed to the corresponding author/s.

## Ethics Statement

The studies involving human participants were reviewed and approved by the Institutional Research Ethics Committee of Guizhou Provincial People’s Hospital. The patients/participants provided their written informed consent to participate in this study.

## Author Contributions

PZ was responsible for the conception and design of bioinformatic analysis, experiments, and providing experimental funds. QY was responsible for the original draft preparation, data curation, and statistical analysis. Both authors have read and approved the final manuscript.

## Conflict of Interest

The authors declare that the research was conducted in the absence of any commercial or financial relationships that could be construed as a potential conflict of interest.

## Footnotes

1 https://www.tnmplot.com/

2 https://www.oncomine.org/

3 https://portal.gdc.cancer.gov/

4 http://gepia.cancer-pku.cn/

5 http://www.prognoscan.org/

6 https://string-db.org/

7 http://metascape.org/gp/index.html#/main/

## Supplementary Material

Supplementary Figure 1 Genes co-expressed with SHMT2 in BLCA cells SHMT2 is strong co-expressed with the indicated genes in BLCA cells (Lee Cell Line 2) (Pearson correlation coefficient > 0.6). The bar length represents the significance and negative logarithm of the enrichment p value.

Supplementary Figure 2 The protein-protein interaction network of SHMT2 The PPI network that was closely associated with SHMT2.

Supplementary Table 1 Baseline data of bladder cancer patients of TCGA.

Supplementary Table 2 Genes co-expressed with SHMT2 from the Oncomine database (Lee Celline 2).

Supplementary Table 3 KEGG pathway enrichment in BLAC with SHMT2 high expression.
