# Supplementary figures and images for: Overexpression of SHMT2 Predicts a Poor Prognosis and Promotes Tumor Cell Growth in Bladder Cancer
Source: Front Genet. 2021 Jun 4;12:682856. doi: 10.3389/fgene.2021.682856 (PMC8212063; doi:10.3389/fgene.2021.682856)

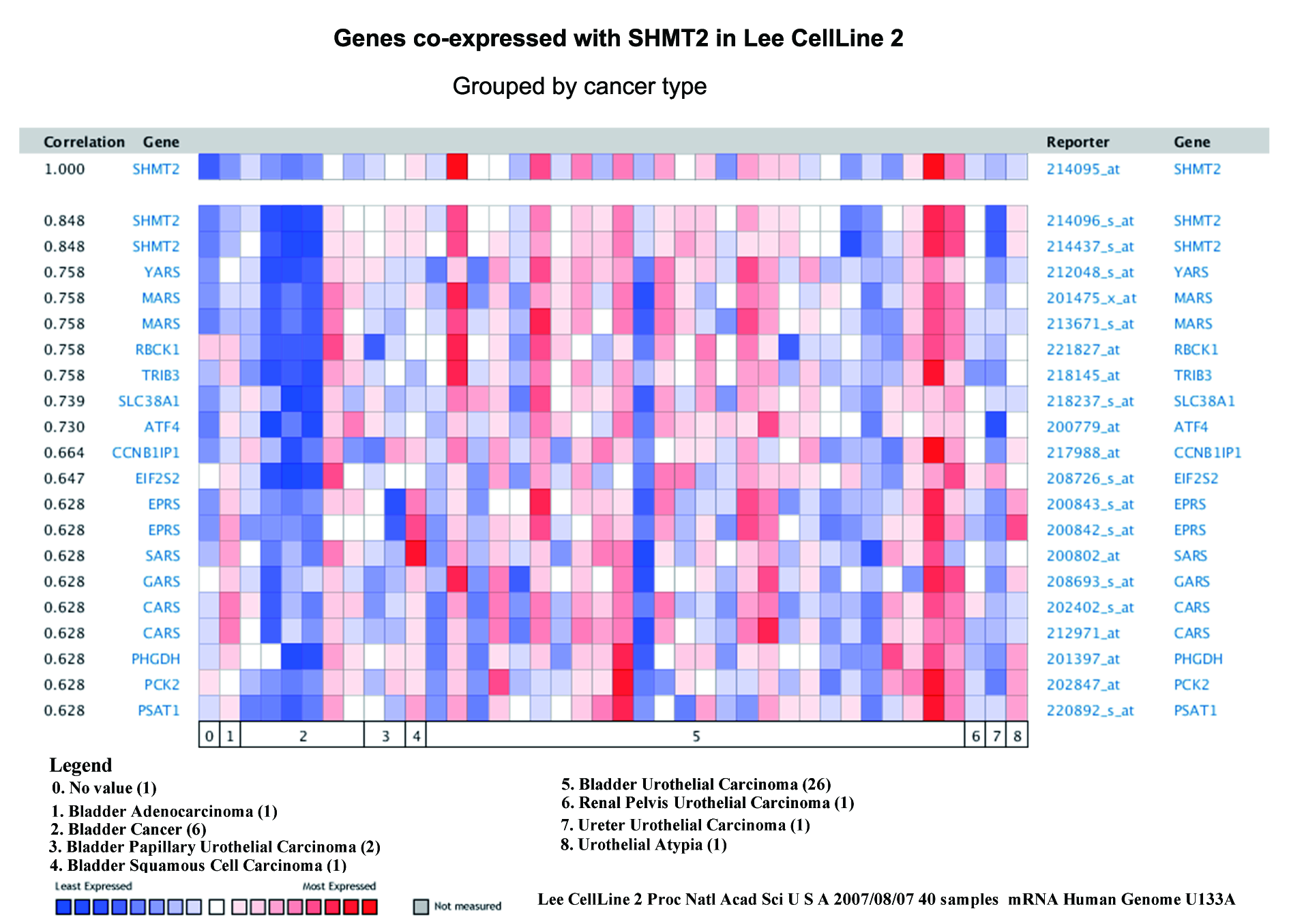

Supplement: Supplementary Figure 1 — Genes co-expressed with SHMT2 in BLCA cells SHMT2 is strong co-expressed with the indicated genes in BLCA cells (Lee Cell Line 2) (Pearson correlation coefficient > 0.6). The bar length represents the significance and negative logarithm of the enrichment p value. [file Image_1.TIF]

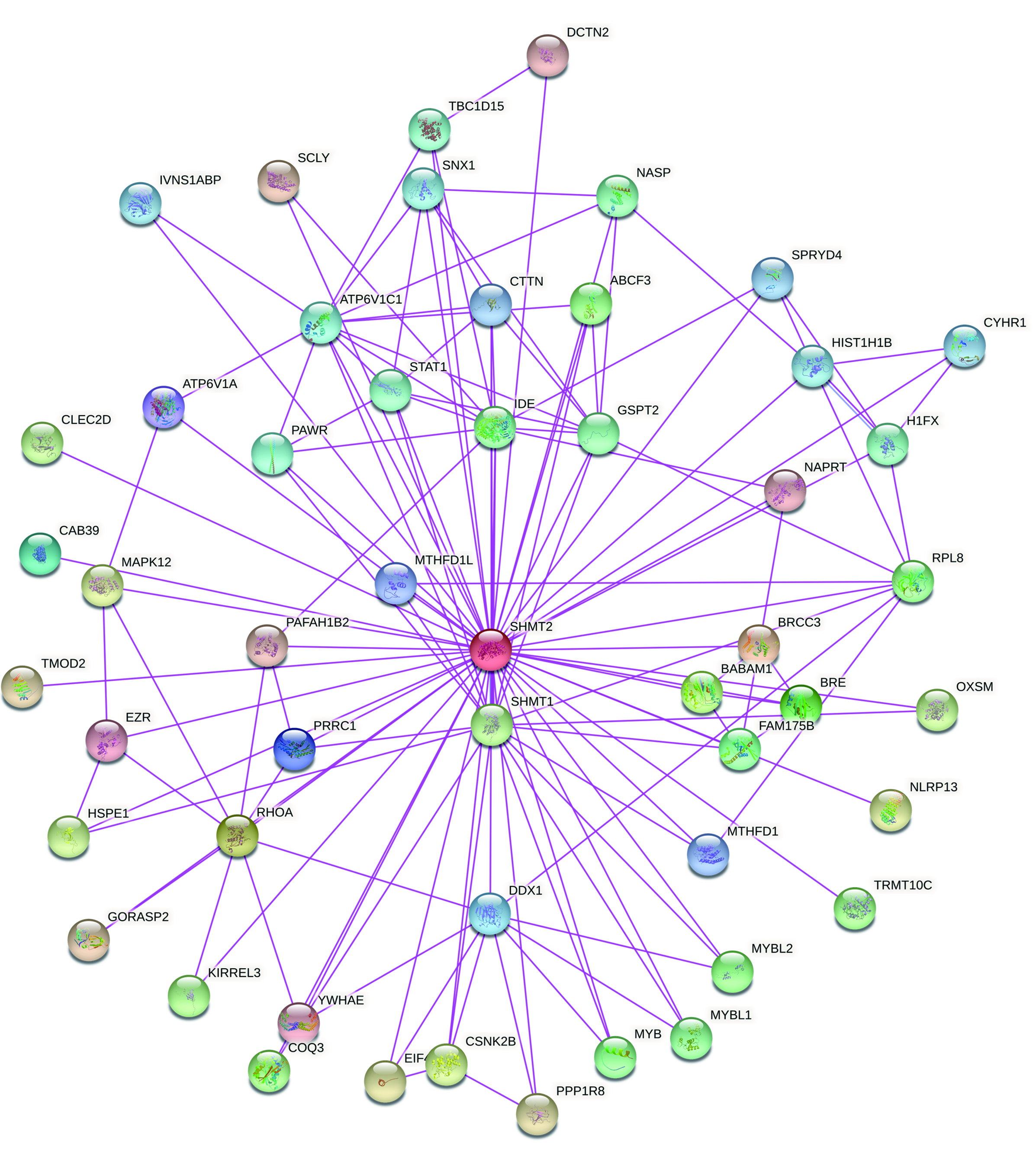

Supplement: Supplementary Figure 2 — The protein-protein interaction network of SHMT2 The PPI network that was closely associated with SHMT2. [file Image_2.TIF]
