# Supplementary material for: Overexpression of SHMT2 Predicts a Poor Prognosis and Promotes Tumor Cell Growth in Bladder Cancer
Source: Front Genet. 2021 Jun 4;12:682856. doi: 10.3389/fgene.2021.682856 (PMC8212063; doi:10.3389/fgene.2021.682856)
Supplement: Supplementary Table 1 — Baseline data of bladder cancer patients of TCGA. [file Table_1.DOC]

**Supplementary Table 1 Baseline data of bladder cancer patients of TCGA**

| Clinicopathological parameters | Total = 408 | |
| --- | --- | --- |
| **Age (years)** | **N** | % |
| ≤60 | 107 | 26.23 |
| >60 | 301 | 73.77 |
| **Gender** |  |  |
| Female | 107 | 26.23 |
| Male | 301 | 73.77 |
| **Grade** |  |  |
| High | 384 | 94.12 |
| Low | 21 | 5.15 |
| Unknown | 3 | 0.74 |
| **T stage** |  |  |
| T1+T2 | 123 | 30.15 |
| T3+T4 | 252 | 61.76 |
| Unknown | 33 | 8.09 |
| **N stage** |  |  |
| N0 | 237 | 58.09 |
| N1-N3 | 129 | 31.62 |
| Unknown | 42 | 10.29 |
| **M stage** |  |  |
| M0 | 196 | 48.04 |
| M1 | 11 | 2.70 |
| Unknown | 201 | 49.26 |
| **TNM stage** |  |  |
| I +II | 132 | 32.35 |
| III+ IV | 274 | 67.16 |
| Unknown | 2 | 0.49 |
| **Survival status** |  |  |
| Dead | 156 | 38.24 |
| Living | 251 | 61.52 |
| Unknown | 1 | 0.25 |
