# Supplementary material for: Overexpression of SHMT2 Predicts a Poor Prognosis and Promotes Tumor Cell Growth in Bladder Cancer
Source: Front Genet. 2021 Jun 4;12:682856. doi: 10.3389/fgene.2021.682856 (PMC8212063; doi:10.3389/fgene.2021.682856)
Supplement: Supplementary Table 2 — Genes co-expressed with SHMT2 from the Oncomine database (Lee Celline 2). [file Table_2.DOC]

**Supplementary Table 2** Genes co-expressed with SHMT2 from the Oncomine database (Lee Celline 2)

| Gene | Description | Correlation coefficient |
| --- | --- | --- |
| SHMT2 | Serine hydroxymethyltransferase | 1 |
| YARS | Tyrosine-tRNA ligase | 0.758 |
| MARS | Methionine-tRNA ligase | 0.758 |
| RBCK1 | RanBP-type and C3HC4-type zinc finger-containing protein 1 | 0.758 |
| TRIB3 | Tribbles homologue | 0.758 |
| SLC38A1 | Sodium-coupled neutral amino acid transporter 1 | 0.739 |
| ATF4 | Cyclic AMP-dependent transcription factor ATF-4 | 0.730 |
| EIF2S2 | Eukaryotic translation initiation factor 2 subunit 2 | 0.647 |
| EPRS | Bifunctional glutamate/proline--tRNA ligase | 0.628 |
| SARS | Serine-tRNA ligase | 0.628 |
| GARS | Glycine-tRNA ligase | 0.628 |
| CARS | Cysteine-tRNA ligase | 0.628 |
| PHGDH | D-3-phosphoglycerate dehydrogenase | 0.628 |
| PCK2 | Phosphoenolpyruvate carboxykinase [GTP] | 0.628 |
| PSAT1 | Phosphoserine aminotransferase | 0.628 |
