# Supplementary material for: Overexpression of SHMT2 Predicts a Poor Prognosis and Promotes Tumor Cell Growth in Bladder Cancer
Source: Front Genet. 2021 Jun 4;12:682856. doi: 10.3389/fgene.2021.682856 (PMC8212063; doi:10.3389/fgene.2021.682856)
Supplement: Supplementary Table 3 — KEGG pathway enrichment in BLAC with SHMT2 high expression. [file Table_3.DOCX]

**Supplementary table 3** KEGG pathway enrichment in BLAC with SHMT2 high expression

| Name | Size | ES | NES | NOM *p*-value | FDR q-value |
| --- | --- | --- | --- | --- | --- |
| KEGG_Cell cycle | 124 | 0.757 | 2.39 | 0.00 | 0 |
| KEGG_Spliceosome | 127 | 0.799 | 2.363 | 0.00 | 0 |
| KEGG_Oocyte meiosis | 112 | 0.616 | 2.296 | 0.00 | 1.65E-04 |
| KEGG_Purine metabolism | 157 | 0.569 | 2.249 | 0.00 | 2.89E-04 |

**Note:** ES: Enrichment Score; NES: normalized enrichment score; NOM: nominal; FDR: false discovery rate; NOM *p*-value <0.05 and FDR q-value <0.05 were recognized as significant.
